# Haploidentical donor transplant is associated with secondary poor graft function after allogeneic stem cell transplantation: A single‐center retrospective study

**DOI:** 10.1002/cam4.4353

**Published:** 2021-10-20

**Authors:** Wei‐Ran Lv, Ya Zhou, Jun Xu, Zhi‐Ping Fan, Fen Huang, Na Xu, Li Xuan, Peng‐Cheng Shi, Hui Liu, Zhi‐Xiang Wang, Jing Sun, Qi‐Fa Liu

**Affiliations:** ^1^ Department of Hematology Nanfang Hospital Southern Medical University Guangzhou China

**Keywords:** haploidentical donor transplant, hazard elements, hematopoietic stem cell transplantation, secondary poor graft function

## Abstract

**Background:**

Secondary poor graft function (sPGF) is a serious complication after allogeneic hematopoietic stem cell transplantation (allo‐HSCT) related to poor outcome. We aimed to retrospectively evaluate the morbidity and hazard elements of sPGF after allo‐HSCT.

**Methods:**

Eight hundred and sixty‐three patients who achieved initial engraftment of both neutrophils and platelets were retrospectively reviewed in this study.

**Results:**

Fifty‐two patients developed sPGF within 180 days post‐transplants, with the median onset time was 62 days (range, 34–121 days) post‐transplants. The overall cumulative incidence of sPGF within 180 days post‐transplantation was 6.0%, with 3.4%, 3.4%, and 10.1%, respectively, in matched sibling donor (MSD), matched unrelated donor (MUD), and haploidentical donor (HID) transplant (*p* < 0.0001). Multivariable analysis showed that HID (HID vs. MSD: hazard ratio [HR] 2.525, *p* = 0.004; HID vs. MUD: [HR] 3.531, *p* = 0.017), acute graft versus host disease (aGVHD) within +30 days ([HR] 2.323, *p* = 0.003), and cytomegalovirus (CMV) reactivation ([HR] 8.915, *p* < 0.0001) within +30 days post‐transplants were hazard elements of sPGF. The patients with sPGF had poorer survival than good graft function (51.7±8.1% vs. 62.9±1.9%, *p* < 0.0001). Our results also showed that only CMV reactivation was the hazard element for the development of PGF in HID transplant ([HR] 12.521 *p* < 0.0001).

**Conclusion:**

HID transplant is also an independent hazard element of sPGF except for aGVHD and CMV reactivation.

## Graphical abstract

Except for acute graft versus host disease and cytomegalovirus (CMV) reactivation, haploidentical donor (HID) transplant is also an independent risk factor for secondary poor graft function (sPGF). The high risk of sPGF in HID transplant might be associated with their high incidence of CMV reactivation.

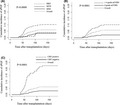

## 1 INTRODUCTION

Complete and stable hematopoietic reconstitution is a key element of allogeneic hematopoietic stem cell transplantation (allo‐HSCT) success.^1^, ^2^ The occurrence of initial hematopoiesis engraftment is generally within 4 weeks post‐transplant.^3^ The recipients who fail to achieve the initial engraftment or lose their initial hematopoietic reconstitution are defined as graft failure, which can be classified into poor graft function (PGF) and graft rejection.^4^, ^5^, ^6^ PGF is a serious complicating disease after allo‐HSCT, leading to high mortality.^7^, ^8^, ^9^, ^10^ Generally, PGF is divided into primary PGF, which fails to achieve initial hematopoietic reconstitution, and secondary PGF (sPGF), which loses initial hematopoietic reconstitution.^11^, ^12^ In clinic, sPGF is more frequent than primary PGF, with the incidence of 5%–27%.^7^, ^8^, ^9^, ^10^ Many factors have been demonstrated to be related to sPGF development, such as graft versus host disease (GVHD), virus infections, and so on.^5^, ^6^, ^7^ However, whether haploidentical donor (HID) transplant is a hazard element of sPGF remains unclear.

In our research, we retrospectively analyzed the morbidity, hazard elements, and outcome in patients with sPGF after allo‐HSCT. Our result suggested that transplantation from HID was a hazard element of sPGF.

## 2 PATIENTS AND METHODS

Adult patients with hematological malignancies who received their initial transplantation were retrospectively reviewed between 1 January 2014 and 30 June 2019 at our institution. The patients who obtained initial neutrophils (NEUs) engraftment and platelets (PLTs) engraftment as well as a fully chimeric state by the +30 day after receiving transplantation were reviewed, and the patients undergoing non‐myeloablative transplantation were excluded from this study. Protocol of our research was performed according to the Declaration of Helsinki. Our institution also approved this research according to Review Board.

### 2.1 Transplant procedures

Donor selection, conditioning regimen, GVHD, and infection prophylaxis were described in our previous reports.^3^, ^4^, ^5^, ^6^, ^7^, ^8^, ^9^, ^10^, ^11^, ^12^, ^13^, ^14^, ^15^, ^16^ HLA‐matched sibling donor (MSD) was priority. In the absence of MSD or a suitable matched unrelated donor (MUD), HID would be chosen. Mixed grafts of bone marrow (BM) stem cells and peripheral blood stem cells (PBSCs) were transplanted into HID patients, whereas PBSCs grafts were transplanted into MSD and MUD patients. Five myeloablative conditioning regimens were used in our center as described previously.^14^, ^15^ The regimens included TBI (total body irradiation, 4.5 Gy/day, −5, −4 days) + Cy (cyclophosphamide, 60 mg/kg/day, −3, −2 days), Bu (busulfan, 3.2 mg/kg/day, −6 to −3 days) + Cy (60 mg/kg/day, −3, −2 days), Bu (3.2 mg/kg/day, −6 to −3 days) + Flu (fludarabine, 30 mg/m^2^, −6 to −2 days), intensified myeloablative conditioning (TBI [4.5 Gy/day, −5, −4 days] + CY + VP‐16 [etoposide, 10–15 mg/kg/day, −3, −2 days]), and sequential intensified conditioning (Flu [30 mg/m^2^/day, −10 to −6 days] + Ara‐C [cytarabine, 2.0 g/m^2^/day, −10 to −6 days] plus TBI [4.5 Gy/day, −5, −4 days]). Generally, acute myeloid leukemia (AML) in complete response (CR) received BuCy or BuF, and acute lymphoid leukemia (ALL) received TBI + Cy or TBI + Cy + etoposide, and acute biphenotypic leukemia or whose diseases were in no response (NR) received sequential intensified preparative regimen. MSD patients received cyclosporin A (CsA) + methotrexate (MTX) to guard against the occurrence of GVHD. MUD and HID recipients received CsA + MTX + antithymocyte globulin (ATG) and/or mycophenolate mofetil to prevent GVHD.^14^ Total ATG doses of 7.5 mg/kg, on days −3 to −1 were used in MUD recipients while total ATG doses of 10 mg/kg, on days −3 to 0 were used in HID recipients. Infection prophylaxis was in accordance with previous literature. Patients with cytomegalovirus (CMV) or EBV‐emia achieved preemptive therapy.^15^, ^16^

### 2.2 Evaluation points and definitions

Our research mainly explored the morbidity and hazard elements of sPGF. Reconstruction of NEUs was defined as in the absence of stimulating by granulocyte colony‐stimulating factor (G‐CSF) at the first 3 successive days post‐transplantation, the absolute NEU number could achieve 0.5 × 10^9^/L. Reconstitution of PLTs definition was that PLT number was ≥20 × 10^9^/L without PLT infusion at the first 7 successive days after receiving transplantation. The recipients who achieved consistent reconstitution of both NEUs and PLTs with no need for transfusion were defined as good graft function (GGF). sPGF definition was that sustained neutropenia (NEU count ≤0.5 × 10^9^/L), thrombocytopenia (PLT count ≤20 × 10^9^/L), and/or hemoglobin (Hb) ≤70 g/L for a minimum of 3 successive days with complete donor chimerism, or depending on requirements of G‐CSF support and/or blood transfusion after day +30 post‐HSCT.^11^, ^12^, ^17^ In addition, patients with serious GVHD or hematological recurrence were removed from sPGF diagnosis.^11^, ^12^, ^17^ Hematological recurrence definition is that the tumor cells appeared again in patients’ peripheral blood and the rate of recurrence blasts in BM was greater than 5%. In addition, appearance of extramedullary infiltration at any time also belonged to relapse.^12^, ^13^ Overall survival definition was that time from transplantation to death or date of last follow‐up in alive patients. The response criteria of sPGF were defined as follows: (1) CR: NEUs >1.5 × 10^9^/L and PLTs >50 × 10^9^/L for 3 continuous days posttreatment; (2) Partial response (PR): NEUs >0.5 × 10^9^/L and PLTs >20 × 10^9^/L for 3 continuous days post‐treatment but failed to achieve the diagnostic criteria of CR; (3) NR: did not reach the above two standards; and (4) Overall response: including both CR and PR.

### 2.3 Statistical analysis

Patient follow‐up was up to 30 April 2020. Continuous variables were stated as the median and categorical variables were stated as a percentage (%). One‐way ANOVA was performed for comparison of continuous variables. The Chi‐squared test or Fisher's exact test was performed for comparison of percentage. Survival rate was analyzed by life table method. The Kaplan–Meier method was performed to analyze survival, nonrecurrent mortality, and cumulative incidence of CMV reactivation within +30 days. For comparison between different groups, the log‐rank test (Mantel–Haenszel) was applied. Multivariate analysis used Cox regression to further evaluate the hazard elements. Cumulative incidence was applied to calculate the incidence of sPGF and the death was seen as a risk of competition. Cumulative incidence was also performed to calculate NEUs and PLTs response the death before response was seen as a risk of competition. Two‐sided *p* values were applied. A *p* < 0.05 was regarded as statistical significance. SPSS Version 19.0 was applied to analyze the statistical data. Competitive risk model in R method (R version 3.4.3) was used to analyze the cumulative incidence.

## 3 RESULTS

### 3.1 Patient demographics and transplants characteristics

In all, 998 patients with malignant hematological diseases were reviewed in current retrospective research. Finally, 863 patients were reviewed while 125 were excluded due to early death or failing to achieve initial NEUs and PLTs engraftment by the day +30 post‐transplants. Of the 863 patients, 413 underwent MSD, 114 MUD, and 336 HID transplants. Five hundred and fifteen males and 348 females were reviewed in our research and their median age was 32 years (range: 16–60). Primary diseases included AML (*N* = 406), ALL (*N* = 327), myelodysplastic syndromes (*N* = 89), and other hematological malignancies (*N* = 41). Based on the development of sPGF post‐transplants, the reviewed patients were divided into sPGF group and GGF group (Table 1). Transplant characteristics between the two groups are shown in Table 1. Donor source (*p* < 0.0001), HLA disparity (*p* = 0.001), use of ATG (*p* = 0.009), acute graft versus host disease (aGVHD) within +30 days post‐transplants (grades 1–4, *p* = 0.009), CMV reactivation within +30 days post‐transplants (*p* < 0.0001), and EBV reactivation (*p* = 0.032) within +30 days post‐transplants were significantly different between the two groups.

**TABLE 1:** Patients and transplant characteristics

Variate	sPGF (*N* = 52)	GGF (*N* = 811)	*p* value
Recipient sex			0.764
Male	30 (57.7%)	485 (59.8%)	
Female	22 (42.3%)	326 (40.2%)	
Recipient age (years)			0.063
<Median (50)	43 (82.7%)	735 (85.7%)	
≥Median (50)	9 (17.3%)	76 (14.3%)	
Disease			0.974
AML	24 (46.2%)	382 (47.1%)	
ALL	21 (40.4%)	306 (37.7%)	
MDS	5 (9.6%)	84 (10.4%)	
Others	2 (3.8%)	39 (4.8%)	
Donor sex			0.192
Male	28 (53.8%)	510 (62.9%)	
Female	24 (46.2%)	301 (37.1%)	
Donor age (years)			0.642
<Median (50)	7 (13.5%)	719 (88.7%)	
≥Median (50)	45(86.5%)	92 (11.3%)	
Disease status			0.639
CR	38 (73.1%)	616 (76.0%)	
Non‐CR	14 (26.9%)	195 (24.0%)	
Donor source			**<0.0001**
MSD	14 (26.9%)	399 (49.2%)	
MUD	4 (7.7%)	110 (13.6%)	
HID	34 (65.4%)	302 (37.2%)	
Matched HLA loci			**0.001**
Identical	18 (34.6%)	509 (62.8%)	
Three mismatch	6 (11.5%)	55 (6.8%)	
Four mismatch	9 (17.3%)	87 (10.7%)	
Five mismatch	19 (36.6%)	160 (19.3%)	
Blood type			0.688
Match	32 (61.6%)	450 (55.5%)	
Major mismatch	10 (19.2%)	174 (21.5%)	
Minor mismatch	10 (19.2%)	187 (23.0%)	
MNC in graft			0.191
<Median (8.5 × 10^8^/kg)	22(44.3%)	419 (51.7%)	
≥Median (8.5 × 10^8^/kg)	30 (55.7%)	392 (48.3%)	
CD34^+^ cells in graft			0.386
<Median (2.32 × 10^6^/kg)	23 (44.2%)	409 (50.4%)	
≥Median (2.32 × 10^6^/kg)	29 (55.8%)	402 (49.6%)	
WBC engraft (days)			0.484
<Median (13)	23(44.2%)	319(39.3%)	
≥Median (13)	29(55.8%)	492(60.7%)	
PLT engraft (days)			0.269
<Median (14)	19(36.5%)	360(44.4%)	
≥Median (14)	33(63.5%)	451(55.6%)	
GVHD prophylaxis			**0.009**
ATG	38 (73.1%)	441 (54.4%)	
Non‐ATG	14 (26.9%)	370 (45.6%)	
aGVHD (within +30 days)			**0.009**
Grades 1–4	30 (57.7%)	319 (39.3%)	
Grade 0	22 (42.3%)	492 (60.7%)	
CMV reactivation (within +30 days)			**<0.0001**
Positive	31 (59.6%)	107 (13.2%)	
Negative	21 (40.4%)	704 (86.8%)	
EBV reactivation (within +30 days)			**0.032**
Positive	23 (44.2%)	244 (30.1%)	
Negative	29 (55.8%)	567 (69.9%)	

Abbreviations: aGVHD, acute graft versus host disease; ALL, acute lymphoid leukemia; AML, acute myeloid leukemia; ATG, antithymocyte globulin; CMV, cytomegalovirus; CR, complete remission; EBV, Epstein–Barr virus; GGF, good graft function; GVHD, graft versus host disease; HID, haploidentical‐related donor; HLA, human leukocyte antigen; MDS, myelodysplastic syndromes; MNC, mononuclear cell; MSD, matched sibling donor; MUD, matched unrelated donor; Others, include acute undifferentiated leukemia, chronic myeloid leukemia and lymphoma; PLT, platelet; sPGF, secondary poor graft function; WBC, white blood count.

*p* < 0.05 was shown in bold.

### 3.2 Incidence and hazard elements of sPGF within 180 days post‐transplants

**FIGURE 1:**
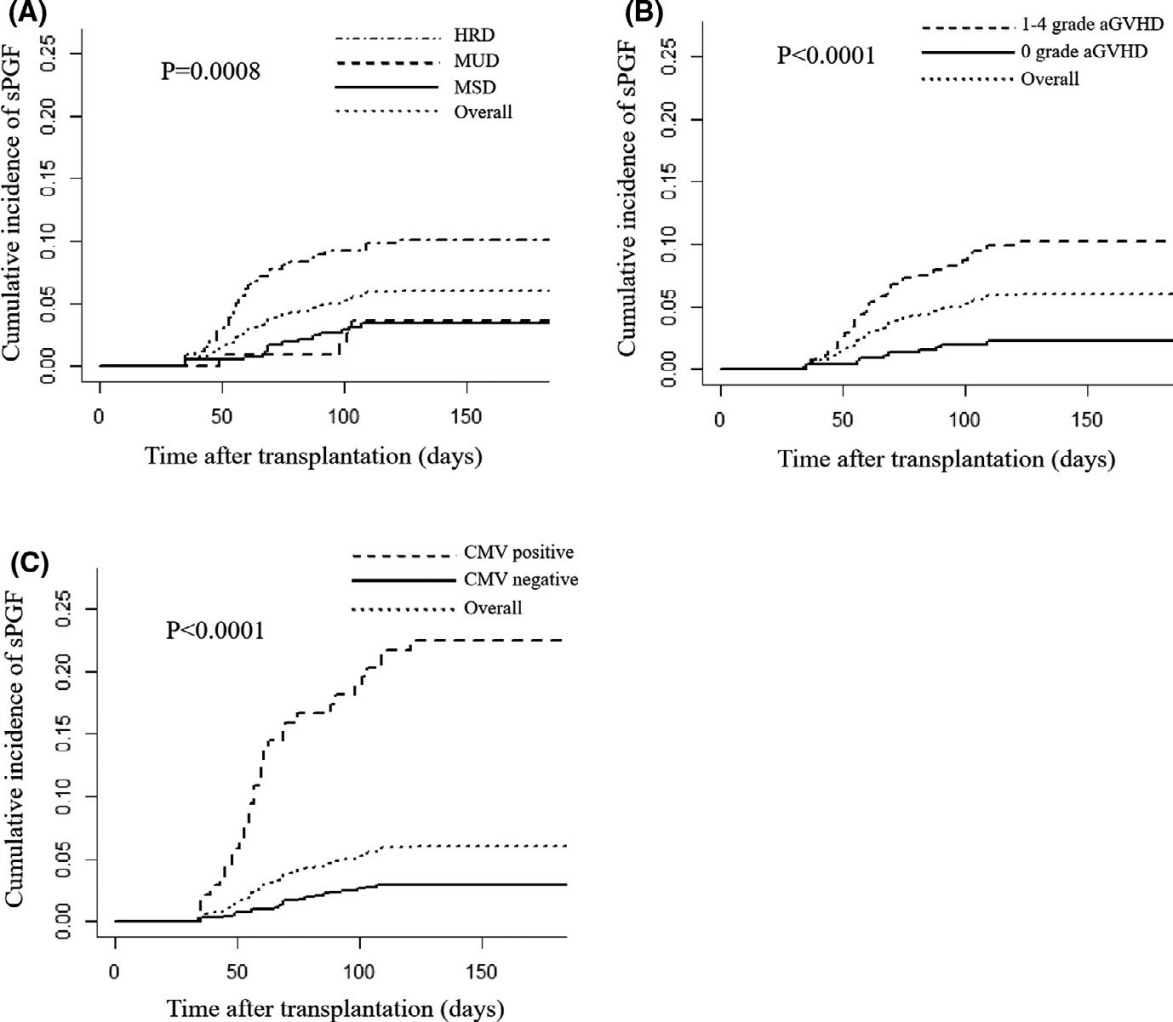
Cumulative incidence of sPGF. (A) Cumulative incidence of sPGF within 180 days post‐transplants according to type of donor. (B) Cumulative incidence of sPGF on 180 days post‐transplants according to occurrence of aGVHD. (C) Cumulative incidence of sPGF within 180 days post‐transplants according to CMV reactivation. aGVHD, acute graft versus host disease; CMV, cytomegalovirus; sPGF, secondary poor graft function

Among the 863 patients reviewed, 52 developed sPGF within 180 days post‐transplants, with along the median time of sPGF onset was 62 days (range, 34–121 days) post‐transplants. Thirty males and 22 females occurred sPGF and their median age at transplants was 33 years (range: 16–55). The overall cumulative incidence of sPGF within 180 days post‐transplants was 6.0% (95% confidence interval [CI]: 4.58%–7.77%) (Figure 1), with 3.4% (95% CI: 1.94%–5.47%), 3.4% (95% CI: 1.16%–8.24%), and 10.1% (95% CI: 7.20%–13.67%), respectively, in MSD, MUD, and HID transplant. Hazard elements of sPGF are shown in Table 2. Univariable analysis showed that recipient age (*p* = 0.044), donor source (*p* < 0.0001), HLA disparity (*p* < 0.0001), use of ATG (*p* = 0.006), aGVHD within +30 days post‐transplants (grades 1–4, *p* = 0.008), CMV reactivation within +30 days post‐transplants (*p* < 0.0001), and EBV reactivation within +30 days post‐transplants (*p* = 0.037) were hazard elements of sPGF. Multivariable analysis showed that HID transplant (HID vs. MSD: *p* = 0.004, hazard ratio [HR] 2.525 [95% CI: 1.349–4.728]; HID vs. MUD: *p* = 0.017, [HR] 3.531 [95% CI: 1.252–9.956]; MSD vs. MUD: *p* = 0.789), aGVHD within +30 days post‐transplants (grades 1–4, *p* = 0.003, [HR] 2.323 [95% CI: 1.335–4.043]), and CMV reactivation (*p* < 0.0001, [HR] 8.915 [95% CI: 5.100–15.985]) were identified as independent hazard elements of sPGF.

**TABLE 2:** Univariable and multivariable analyses for hazard elements of sPGF

Variable	Univariate	Multivariate (HR)
Recipient sex	*p* = 0.649	—
Male		
Female		
Recipient age (years)	***p* = 0.044**	—
<Median (50)		
≥Median (50)		
Disease	*p* = 0.977	—
AML		
ALL		
MDS		
Others		
Donor sex	*p* = 0.202	—
Male		
Female		
Donor age (years)	*p* = 0.683	—
<Median (50)		
≥Median (50)		
Disease status	*p* = 0.488	—
CR		
Non‐CR		
Donor source	***p* < 0.0001**	
MSD		**HID versus MSD: *p* = 0.004 (2.525) 95% CI: 1.349–4.728**
MUD		**HID versus MUD: *p* = 0.017 (3.531) 95% CI: 1.252–9.956**
HID		
Matched HLA loci	***p* < 0.0001**	—
Identical		
Three mismatch		
Four mismatch		
Five mismatch		
Blood type	*p* = 0.716	—
Match		
Major mismatch		
Minor mismatch		
MNC in graft	*p* = 0.228	—
<Median (10 × 10^8^/kg)		
≥Median (10 × 10^8^/kg)		
CD34^+^ cells in graft	*p* = 0.354	—
<Median (2.32 × 10^6^/kg)		
≥Median (2.32 × 10^6^/kg)		
WBC engraft (days)	*p* = 0.474	—
<Median (13)		
≥Median (13)		
PLT engraft (days)	*p* = 0.319	—
<Median (14)		
≥Median (14)		
GVHD prophylaxis	***p* = 0.006**	—
ATG		
Non‐ATG		
aGVHD (within +30 days)	***p* = 0.008**	***p* = 0.003(2.323)**
Grades 1–4		**95% CI: 1.335–4.043**
Grade 0		
CMV reactivation (within +30 days)	***p* < 0.0001**	***p* < 0.0001(8.915)**
Positive		**95% CI: 5.100–15.985**
Negative		
EBV reactivation (within +30 days)	***p* = 0.037**	—
Positive		
Negative		

Abbreviations: aGVHD, acute GVHD; ALL, acute lymphoid leukemia; AML, acute myeloid leukemia; ATG, antithymocyte globulin; CI, confidence interval; CMV, cytomegalovirus; CR, complete remission; EBV, Epstein–Barr virus; GVHD, graft versus host disease; HID, haploidentical‐related donor; HLA, human leukocyte antigen; HR, hazard ratio; MDS, myelodysplastic syndromes; MNC, mononuclear cell; MSD, matched sibling donor; MUD, matched unrelated donor; Others, include acute undifferentiated leukemia, chronic myeloid leukemia and lymphoma; PLT, platelet; sPGF, secondary poor graft function; WBC, white blood count.

The cumulative incidence of sPGF in patients with HID transplant (10.1%, 95% CI: 7.20%–13.67%) was higher than those with MSD (3.4%, 95% CI: 1.94%–5.47%; *p* < 0.0001) and MUD (3.4%, 95% CI: 1.16%–8.24%; *p* = 0.032) transplant (Figure 1A). Cumulative incidence of sPGF in patients with grade 1–4 aGVHD within +30 days post‐transplants was 10.2% (95% CI: 7.49%–13.31%), higher than those without aGVHD 2.2% (95% CI: 1.15%–3.93%; *p* < 0.0001) (Figure 1B). Recipients with CMV reactivation within +30 days post‐transplants (22.5%, 95% CI: 15.91%–25.78%) had higher incidence of sPGF than those without CMV reactivation (2.9%, 95% CI: 1.85%–4.32%; *p* < 0.0001) (Figure 1C).

To explore the reason why HID transplant was a hazard element for PGF, we further analyzed the relation between the development of PGF and CMV reactivation in patients undergoing HID transplantation. Our results showed that only CMV reactivation was the hazard element for the development of PGF in HID transplant (*p* < 0.0001, [HR] 12.521 [95% CI: 5.982–26.209]) (Table 3). In addition, we analyzed the incidence of CMV reactivation within +30 days, CMV serostatus of recipients and donors before transplantation, and levels of maximum viral loads among MRD, MUD, and HID. Our results showed that the cumulative incidence of CMV reactivation within +30 days of HID (19.0±2.1%) was higher than MSD (14.5±1.7%, *p* = 0.023) and MUD (12.3±3.1%, *p* = 0.035), while there was no significant difference between MSD and MUD (*p* = 0.367). CMV serostatus (CMV IgG) of recipients and donors before transplantation was not significantly different among MSD, MUD, and HID (data not shown). The levels of median viral loads within +30 days in MSD, MUD, and HID were 8.02 × 10^4^ copies/ml (range, 0.12–17.60 × 10^4^ copies/ml), 12.48 × 10^4^ copies/ml (range, 0.23–22.31 × 10^4^ copies/ml), and 17.75 × 10^5^ copies/ml (range, 0.07–85.60 × 10^4^ copies/ml), respectively. The viral loads within +30 days in HID were higher than MSD (*p* < 0.0001) and MUD (*p* = 0.003), while there was no significant difference between MSD and MUD (*p* = 0.601).

**TABLE 3:** Univariable and multivariable analyses for hazard elements of sPGF in HID transplantation

Variable	Univariate	Multivariate (HR)
Recipient sex	*p* = 0.449	—
Male		
Female		
Recipient age (years)	*p* = 0.868	—
<Median (50)		
≥Median (50)		
Disease	*p* = 0.745	—
AML		
ALL		
MDS		
Others		
Donor sex	*p* = 0.613	—
Male		
Female		
Donor age (years)	*p* = 0.965	—
<Median (50)		
≥Median (50)		
Disease status	*p* = 0.276	—
CR		
Non‐CR		
Blood type	*p* = 0.114	—
Match		
Major mismatch		
Minor mismatch		
MNC in graft	*p* = 0.511	—
<Median (10 × 10^8^/kg)		
≥Median (10 × 10^8^/kg)		
CD34^+^ cells in graft	*p* = 0.663	—
<Median (2.41 × 10^6^/kg)		
≥Median (2.41 × 10^6^/kg)		
WBC engraft (days)	*p* = 0.212	—
<Median (13)		
≥Median (13)		
PLT engraft (days)	*p* = 0.859	—
<Median (14)		
≥Median (14)		
aGVHD (within +30 days)	*p* = 0.172	
Grades 1–4		
Grade 0		
CMV reactivation (within +30 days)	*p* < 0.0001	*p* < 0.0001 (12.521)
Positive		95% CI: 5.982–26.209
Negative		
EBV reactivation (within +30 days)	*p* = 0.988	—
Positive		
Negative		

Abbreviations: aGVHD, acute GVHD; ALL, acute lymphoid leukemia; AML, acute myeloid leukemia; CI, confidence interval; CMV, cytomegalovirus; CR, complete remission; EBV, Epstein–Barr virus; HID, haploidentical‐related donor; HR, hazard ratio; MDS, myelodysplastic syndromes; MNC, mononuclear cell; Others, include acute undifferentiated leukemia, chronic myeloid leukemia and lymphoma; PLT, platelet; sPGF, secondary poor graft function; WBC, white blood count.

### 3.3 Treatment and response

**FIGURE 2:**
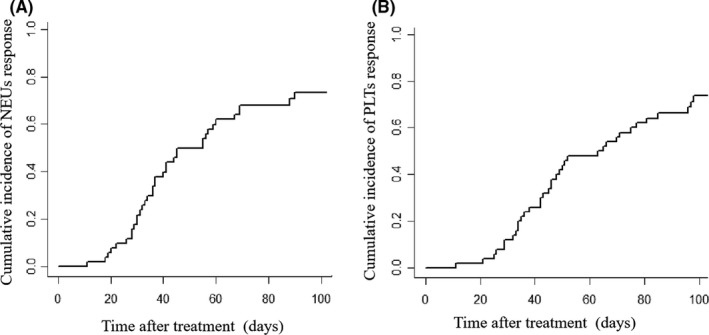
Cumulative incidence of response. (A) Cumulative incidence of NEU response at 100 days after treatments. (B) Cumulative incidence of PLT response at 100 days after treatments. NEU, neutrophil; PLT, platelet

All of the patients with sPGF received supportive treatments, including G‐CSF, thrombopoietin, and transfusion. In addition, 41 patients received special treatments, including 11 receiving PBSCs combining with mesenchymal stem cells (MSCs), 23 cord blood stem cells (UBCs) combining with MSCs, and 7 decitabine. Among them, 37 (71.2%) had response and their median response time after treatments was 47.0 days (11%–137%). The cumulative incidence of NEUs response at 100 days after treatments was 73.3% (95% CI: 57.47%–84.06%), their median time to response was 37 days (range 11–90) (Figure 2A). The cumulative incidence of PLTs response at 100 days after treatments was 73.6% (95% CI: 57.90%–84.25%) and their median time to response was 46 days (range: 11–98) (Figure 2B).

### 3.4 Survival

**FIGURE 3:**
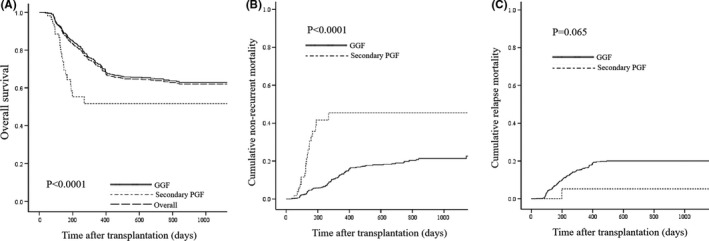
Survival and mortality of sPGF compared with GGF. (A) The 3‐year overall survival of sPGF compared with GGF. (B) The 3‐year cumulative nonrecurrent mortality of sPGF compared with GGF. (C) The 3‐year cumulative relapse mortality of sPGF compared with GGF. GGF, good graft function; sPGF, secondary poor graft function

Of 52 patients with sPGF, 31 survived and 21 died at a median follow‐up of 87.5 days (range, 15–1131 days) after diagnosis of sPGF. The patients with sPGF had poorer 3‐year survival than GGF (51.7±8.1% vs. 62.9±1.9%, *p* < 0.0001) (Figure 3A). Among the 52 patients with sPGF, 2 (3.8%) had relapse of primary malignancy after first engraftment. Among the 811 patients with GGF, 190 (23.4%) had relapsed. The relapse rate of patients with sPGF was lower than patients with GGF (*p* = 0.001). Of 52 patients with sPGF, 1 died of primary disease relapse while 20 died of nonrecurrent mortality factors such as hemorrhage, infection, GVHD, and so on. Of 811 GGF patients who died at the last follow‐up, 141 died of relapse while 125 died of nonrecurrent mortality factors. The 3‐year cumulative nonrecurrent mortality of sPGF patients was higher than GGF patients (45.5±8.0% vs. 21.3±1.0%, *p* < 0.0001, Figure 3B) while the relapse mortality was not significantly different between sPGF and GGF patients (5.3±5.1% vs. 20.0±1.5%, *p* = 0.065, Figure 3C).

## 4 DISCUSSION

PGF is a severe complication that can threaten patients’ life, and the occurrence of sPGF is more frequent than primary PGF.^9^, ^11^, ^12^, ^17^ In our retrospective study, our results proved that the overall cumulative incidence of sPGF within 180 days post‐transplants was 6.0%, with 3.4%, 3.4%, and 10.1%, respectively, in MSD, MUD, and HID transplant. The multivariable analysis showed that hazard elements of sPGF included HID transplant, aGVHD, and CMV reactivation. The patients with sPGF had poorer survival than GGF.

The incidence of sPGF varied from 5% to 27% after allo‐HSCT, depending on the number of hazard elements.^7^, ^8^, ^9^, ^10^ Our results were consistent with the incidence of sPGF reported by Nakamae et al.^5^ and Sun et al.,^11^ in which the incidence of sPGF within the first 100 days post‐transplants was 7.0% and 5.7%, respectively. A report from Korean revealed that 12.7% patients developed sPGF in the recipients within 60 days after allo‐HSCT.^6^ Many factors may be associated with PGF development, such as prior alloimmunization, conditioning regimen, HLA matching, donor type, GVHD, and infections.^9^, ^17^ Our results showed that aGVHD and CMV reactivation were hazard elements of sPGF, which were consistent with literatures.^11^, ^12^, ^18^, ^19^, ^20^ More importantly, HID transplant was also demonstrated as an independent hazard element of sPGF in our research, which was not consistent with Sun et al. reported.^11^ Emerging experimental and clinical evidence suggests that CMV infection is a major cause of sPGF.^11^, ^12^, ^21^, ^22^, ^23^, ^24^ CMV might directly inhibit hematopoiesis by infecting hematopoietic stem cells and BM stromal cells^21^, ^22^, ^23^, ^24^ or indirectly inhibiting hematopoiesis through antiviral drug toxicities.^5^ Some studies suggest that recipients undergoing HID transplant have a higher incidence of CMV reactivation,^25^, ^26^ which were consistent with our result. In addition, our result showed that CMV reactivation was the only hazard element of sPGF development in the patients with HID transplant. Several groups confirmed the clear association between DSA and primary graft failure as well as PGF in HSCT with HLA‐mismatched donors.^27^, ^28^ Regretfully, the data of DSA in our study were incomplete so that DSA were not involved in our analysis. We will try to improve our DSA data in the future. Based on these, we speculated that the high risk of sPGF after HID transplantation might be associated with high incidence of CMV reactivation in HID. But further exploration is needed.

The prognosis of sPGF is very poor. Limited available therapy options for patients with PGF are found, including hematopoietic growth factors and stem cell reinfusion as well as second transplantation. Hematopoietic growth factors are often effective only for short periods of time. Stem cell reinfusion or second transplantation is related to a high rate of risk of transplant‐related mortality.^17^, ^29^, ^30^, ^31^, ^32^ Response rate of PGF reported in the literatures was 35%–85%.^9^, ^33^, ^34^, ^35^ Our previous study^9^ suggested that BM‐derived MSCs from a third‐party donor combined with donor stem cell or cord blood were effective to PGF. In this study, we obtained the similar result to our previous results.^9^ In addition, Han et al. reported that low‐dose decitabine was effective in patients with isolated thrombocytopenia post‐HSCT.^36^ Of the seven patients with sPGF who received decitabine administration in our study, six had response and significant PLT recovery, which agreed with the good efficacy reported by Han et al.^36^

Retrospective single‐center analysis is the main inadequacy of our research. The multicenter studies are required to verify our observations.

In summary, except for aGVHD and CMV reactivation, HID transplant is also an independent hazard element of sPGF. The high risk of sPGF in HID transplant might be associated with their high incidence of CMV reactivation.

## DISCLOSURE

All of the authors have declared that there was no conflict of interest.

## ETHICS STATEMENT

The study protocol was performed according to the Declaration of Helsinki and was approved by the Institutional Review Board of our institution. All patients provided written informed consent.

## 5 CONFLICT OF INTEREST

The authors declare no relevant conflict of interest.

## Data Availability

All data generated or analyzed during this study are included in this published article.
